# The effect of scopolamine on memory and attention: a systematic review and meta-analysis

**DOI:** 10.1192/j.eurpsy.2025.2446

**Published:** 2025-04-08

**Authors:** Cerena Miravalles, Dara M. Cannon, Brian Hallahan

**Affiliations:** 1 Clinical Research Facility, University of Galway, Galway, Ireland; 2 Clinical Neuroimaging Laboratory, Centre for Neuroimaging and Cognitive Genomics, Galway Neuroscience Centre, College of Medicine, Nursing & Health Sciences, University of Galway, Galway, Ireland

**Keywords:** attention, cognition, memory, scopolamine, cholinergic system

## Abstract

**Background:**

Scopolamine is a muscarinic receptor antagonist and is widely utilized as a “memory-loss model.” However, its impact across different memory and attention tasks and using different modes of administration has yet to be clearly evaluated. This systematic review and meta-analysis investigates the effect of scopolamine, across all routes of administration and across different dosages, on memory and attention performance in healthy humans (PROSPERO ID: CRD42024531634).

**Methods:**

Following the Preferred Reporting Items for Systematic Reviews and Meta-Analyses guidelines, we searched (on 20 April 2024) for studies that utilized scopolamine and assessed memory and/or attention. Random-effects meta-analyses were conducted across a range of memory and attention tasks using “Comprehensive Meta-Analysis,” Version 3, to evaluate differential pharmacological effects on cognitive tasks between the scopolamine and placebo groups.

**Results:**

Forty-six studies fulfilled the inclusion and exclusion criteria. Scopolamine negatively impaired performance on all memory tasks (immediate memory, delayed recall, digit span, Buschke selective reminding task, and recognition memory) and led to slower reaction times for three of the five attention tasks examined (choice reaction time, simple reaction time, and rapid visual information processing) compared to placebo. Scopolamine’s negative effect on memory and attention was greater with injectable (e.g., intramuscular, intravenous, and subcutaneous) compared to non-injectable routes of administration (e.g., intranasal, oral, and transdermal).

**Conclusion:**

This study supports the use of scopolamine as a “memory-loss model,” particularly when given by an injectable route of administration. Future clinical trials should evaluate the bioavailability of scopolamine across different routes of administration to ensure therapeutic benefits outweigh any potential adverse cognitive effects.

## Introduction

Scopolamine, also known as hyoscine, is a tropane alkaloid and a nonselective, pan-muscarinic antagonist that acts as an inhibitor at muscarinic cholinergic receptor sites in the parasympathetic nervous system. Muscarinic cholinergic receptors, which recognize the neurotransmitter acetylcholine (ACh), are a family of seven-transmembrane domain receptors consisting of five receptor subtypes (M_1–5_). Positron emission tomography (PET) studies exhibit scopolamine’s ability to occupy muscarinic cholinergic receptors in both human and nonhuman primates, demonstrating scopolamine’s involvement with the central nervous system (CNS) [1, 2]. Scopolamine induces peripheral and central antimuscarinic effects and is utilized for conditions that require decreased parasympathetic activity, including an antiemetic for motion sickness, postoperative nausea, and a sedative before anesthesia. Adverse effects related to anticholinergic activity are generally mild, but can include pupillary dilatation, tachycardia, decreased production of saliva and mucus, urinary retention, and potentially more rare and severe side effects such as hallucinations and delirium.

The cholinergic system in the human CNS comprised projections from the nuclei of the basal forebrain that innervate the hippocampus and most cortical regions, projections from the brainstem to the thalamus, and interneurons in the striatum and nucleus accumbens [3]. Many of these neuroanatomical areas are responsible for cognition, motor function, and affect [4]. Psychiatric disorders, including schizophrenia, and mood disorders, such as major depressive disorder (MDD) and bipolar disorder (BD), have been linked to dysregulation in the cholinergic system and dysfunction of cholinergic muscarinic receptors, specifically the M_1_ and M_4_ receptor for schizophrenia and the M_2_ receptor for BD [5–10]. An increase of ACh in the CNS has been linked to an exacerbation of depressive symptoms and, conversely, a lack of ACh has been linked to (hypo)manic symptoms [11–13]. Consequently, a number of small randomized controlled trials and a recent systematic review and meta-analysis demonstrated that scopolamine induces a rapid antidepressant effect in individuals experiencing a depressive episode in the context of either MDD or BD [14–19]. Potential adverse sequelae of scopolamine, including on various aspects of cognition, would be important to elucidate if scopolamine becomes a more widely used treatment intervention for the management of acute depressive episodes, particularly as such sequelae have not been examined in detail in treatment trials to date.

Scopolamine has additionally been noted in several studies to produce amnestic effects, likely related to its central anticholinergic activity, resulting in its use to induce memory impairment in healthy humans in studies involving a “memory-loss model” and in studies investigating treatments for dementia [20–39]. PET imaging in monkeys demonstrated impairment in working memory after scopolamine administration [2]. Studies that have explored the potential impact of scopolamine on memory and attention have focused predominantly on constructs, such as working, episodic, semantic, implicit, immediate, visual, long-term, or delayed recognition and verbal memory, as well as on retrieval, coding, and storage of information. While several studies have demonstrated amnestic effects, these findings have not been universally demonstrated, with several studies noting no significant impact on either memory [29, 31, 33, 35] or attention tasks [32, 34]. Variability in scopolamine’s effects may reflect individual differences, with CHRM2 genotype influencing inhibitory control and cholinergic pathways, potentially altering sensitivity to scopolamine-induced cognitive impairment [40]. Consequently, scopolamine’s validity as a model for cognitive dysfunction associated with dementia, including Alzheimer’s disease, has been questioned [41].

There are several factors that might influence the putative impact of scopolamine in relation to memory and attention. First, scopolamine can be administered via a range of different routes, all of which have different pharmacokinetic and metabolic profiles (Supplementary Table S1). Parenteral routes of administration, including intravenous (IV), subcutaneous (SC), and intramuscular (IM) routes, may produce more significant cognitive impairments pertaining to memory and attention [42–44], compared to oral (PO) and transdermal (TD) scopolamine administration [23, 45–47]. Second, higher dosages of scopolamine have been noted in some studies to induce more significant cognitive impairments, although there is limited data exploring if dosage across different modes of administration has a differential impact on performance in tasks pertaining to memory and attention [27, 44, 48].

Examining data systematically pertaining to the potential impact of scopolamine across different routes and dosages of administration in relation to a range of cognitive tasks assessing memory and attention will help inform clinicians of the risks and benefits of this medication, particularly given its continued use as a model of cognitive impairment and its potential future use as an agent with rapid antidepressant effects. Consequently, the aim of this systematic review and meta-analysis is to investigate the effects of scopolamine, across different routes of administration and across different dosages, compared to placebo in relation to its impact on a range of memory and attention performance tasks.

## Methods

We conducted a systematic review that adhered to the Preferred Items for Reporting of Systematic Reviews and Meta-Analyses (PRISMA) checklist (Supplementary Table S2) [49] and preregistered our protocol (https://www.crd.york.ac.uk/prospero/display_record.php?RecordID=531634).

### Eligibility criteria

We included human studies of healthy adult participants (≥18 years of age) to identify the impact of scopolamine administration via any mode of administration on cognitive tasks associated with both memory and attention. All included studies had a placebo arm and were written in English. Review articles, protocols, qualitative/case studies, open-label studies, research meeting abstracts, and conference presentations were excluded. In addition, studies including small sample sizes (≤6 individuals per study arm), where the impact of scopolamine was not possible to determine due to the concurrent administration of other study treatment(s) simultaneously, where the cognitive task included was conducted in less than three studies, or where studies were undertaken in unique environments (i.e., space craft and underwater) were excluded.

### Search strategy

A database search was undertaken with no date restrictions applied, using Medline, Embase, PsychINFO, Web of Science, and the Clinical Trials (https://www.clinicaltrials.gov/) database. Relevant reviews and references of the included studies were searched manually to identify additional appropriate studies for this review. The search included the following medical subject key words: “((scopolamine) OR (hyoscine)) AND (cognition) OR (memory) OR (attention) OR (psychomotor) OR (emotion processing) OR (visual learning) OR (recall) OR (amnesia) OR (amnesic)).

Two authors (CM and BH) independently and blindly screened all the titles and abstracts against the eligibility criteria. Full texts of the remaining studies were assessed against the eligibility criteria (CM and BH), with any disagreements resolved through a discussion between these two authors.

### Data extraction

CM extracted data from all the studies on 20 April 2024, with BH acting as a second blind rater. Any disagreements were resolved with discussion, with any unresolved differences discussed with DC. Effect measures, including mean and standard deviations, were reported as recorded by the study authors. Data extraction included relevant outcomes (observed effects of scopolamine on cognitive tasks), study characteristics (design including cognitive tasks employed, population, dose, and route of scopolamine), and clinical characteristics (population, sample size, age, sex, and education level).

### Quality assessment

The Jadad scale [50] was used to assess the reliability and validity of studies. This tool assesses randomization, blinding, and study withdrawals on a 5-point scale. CM and BH independently and blindly completed the Jadad scale for all included studies, with any differences resolved with a discussion between the authors.

We assessed publication bias using funnel plots when 10 or more studies were included in the analysis. Funnel plots visually assess the symmetry of study effect sizes around the overall effect estimate. Symmetry suggests no significant publication bias, whereas asymmetry may indicate potential bias, such as missing studies with nonsignificant results. For analyses with fewer than 10 studies, funnel plots were not used, as fewer studies reduce the statistical power needed to distinguish a true asymmetry from random variation [51].

### Statistical analysis

A meta-analysis was conducted where three or more studies examined the impact of scopolamine compared to placebo for the same cognitive task. Effect sizes were calculated for continuous data by attaining the mean, standard deviations, and sample size of the scopolamine and placebo groups. When standard deviations were not available, these were estimated based on the other statistical parameters reported in the individual study. Standard errors (SEs) were converted to standard deviations as appropriate. When continuous data were not available, we evaluated dichotomous data and calculated the odds ratios, which were converted into Hedge’s *G* effect size statistic (*G*). For studies using multiple arms of the drug and one arm of the placebo (e.g., different scopolamine doses compared with placebo), the “*n*” for the placebo group was divided by the number of strata in the study. Where sufficient data were available (≥3 studies), additional analyses were performed on “injection” (e.g., IV, IM, and SC) compared to “non-injection” (e.g., PO, TD, and intranasal (IN)) routes of administration. Doses were categorized as “high” (≥0.5 mg) or “low” (<0.5 mg). Age analysis grouped participants into “young” (18–40 years, mean age < 30 years) and “old” (>40 years, mean age > 60 years) cohorts.

“Comprehensive Meta-Analysis,” Version 3, evaluated differential medication effects on cognitive tasks between the scopolamine and placebo groups to ascertain the random-model treatment effect size (*G*), 95% confidence intervals (CIs), and SEs for each study [52]. Heterogeneity of interventions was assessed using the Cochrane *Q* and *I*^2^ statistics, with significance determined at *p* < 0.05.

## Results

### Literature search

**Figure 1.**
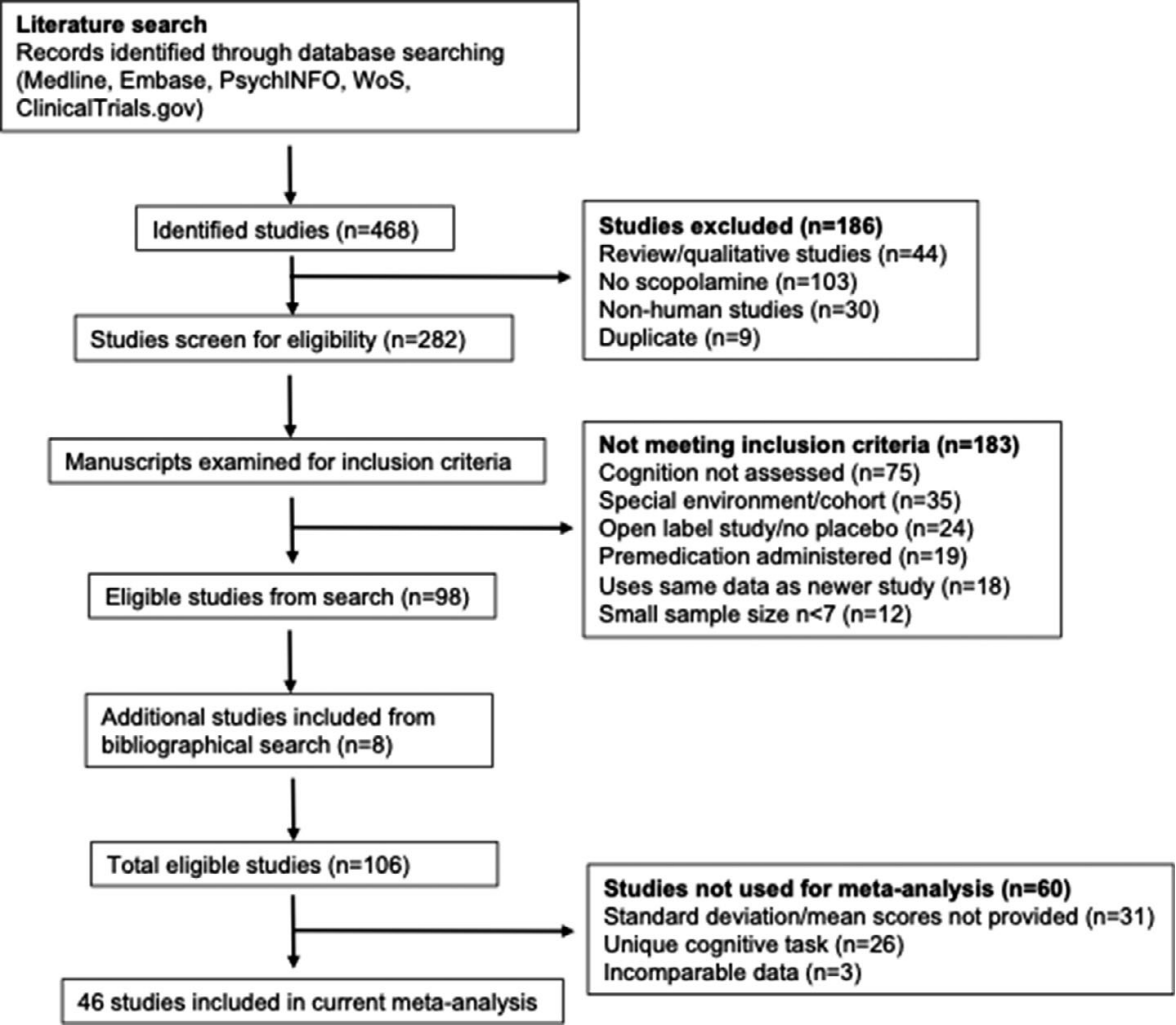
Flowchart describing the study selection process. List of unique cognitive tasks included in Supplementary Table S5.

The PRISMA diagram summarizing the literature search strategy is presented in Figure 1. A total of 468 articles were identified, with 282 full texts reviewed and 106 studies included in the final analysis (eight from the reference lists). Studies were excluded if they lacked cognitive task data, involved open-label designs, used additional treatments, or had intervention arm sizes ≤6 participants. The sociodemographic and clinical characteristics of all included studies are provided in Supplementary Tables S3 and S4.

### Memory

Six different tasks provided data pertaining to performance and reaction time. Scopolamine significantly impaired memory performance and reaction time, only when scopolamine was administered via injection.

#### Free/immediate recall (Figure 2)

Twenty studies (35 strata; scopolamine *n* = 493, placebo *n* = 492) assessed free/immediate recall. Scopolamine impaired accuracy compared to placebo (*G* = −0.86, 95% CI: −1.08 to −0.64, *p* < 0.001), with a significant effect in injection studies (*G* = −1.00, 95% CI: −1.25 to −0.76, *p* < 0.001), but not in non-injection studies (*G* = −0.16, 95% CI: −0.70 to 0.38, *p* = 0.57).

Post- versus pre-administration accuracy was lower in the scopolamine group (*G* = −0.93, 95% CI: −1.42 to −0.44, *p* < 0.001), with insufficient studies present to examine injection and non-injection groups separately (Supplementary Figure S1). Scopolamine impaired performance at both high and low doses, with both the dose categories showing significant effects (Supplementary Figure S2). Evidence of publication or reporting bias, along with heterogeneity among the studies, was observed (Supplementary Figure S3). Performance was assessed 30 min–6 h post-administration, with no discernible impact of timing.

**Figure 2.**
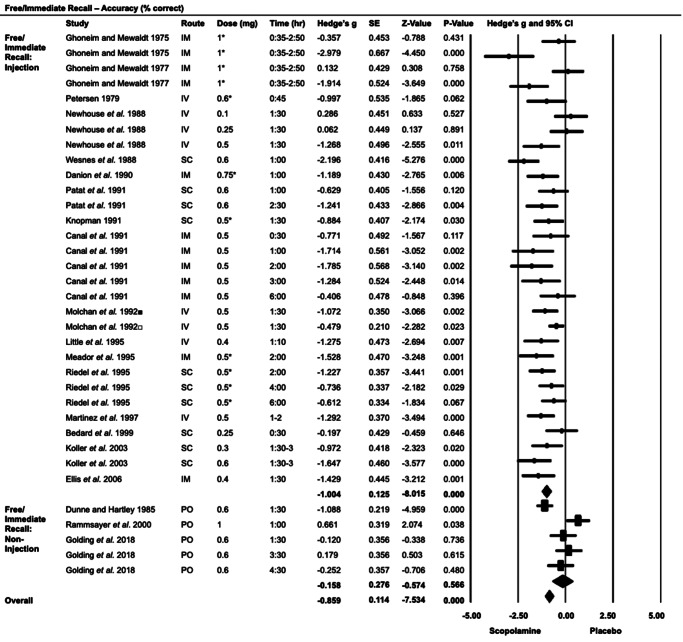
Free/immediate recall: accuracy (% correct). ■ Old cohort. □ Young cohort. IM, intramuscular; IV, intravenous; PO, oral; SC, subcutaneous. *Studies that used microgram doses have been converted to milligrams based on a 75 kg body weight.

#### Delayed recall (Figure 3)

Fourteen studies (21 strata; scopolamine *n* = 332, placebo *n* = 332) utilized delayed recall, with scopolamine impairing performance compared to placebo (*G* = −0.89, 95% CI: −1.16 to −0.61, *p* < 0.001). Both scopolamine injection (*G* = −1.07, 95% CI: −1.41 to −0.72, *p* < 0.001) and non-injection (*G* = −0.56, 95% CI: −1.02 to −0.08, *p* = 0.018) groups performed significantly worse compared to the placebo. Post-administration versus pre-administration accuracy showed no difference (*G* = −0.29, 95% CI: −1.08 to 0.50, *p* = 0.47); however, three of the five strata included non-injectable scopolamine (Supplementary Figure S1). Scopolamine impaired performance at both high and low doses, with both the dose categories showing significant effects (Supplementary Figure S4). Evidence of publication or reporting bias was observed in the delayed recall task assessing performance (Supplementary Figure S5). This task was assessed 30 min–4.5 h post-administration, with no apparent impact of the timing.

**Figure 3.**
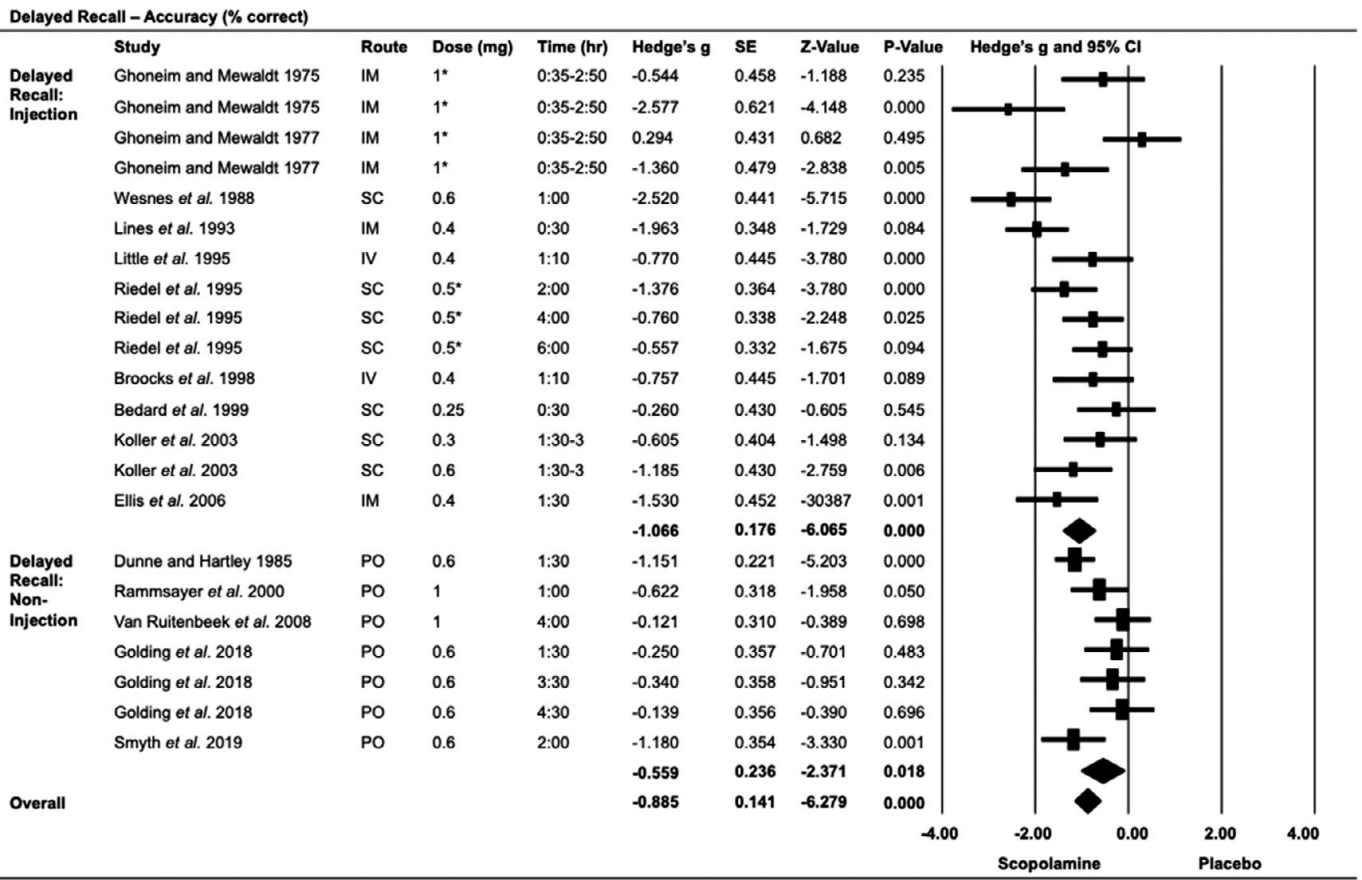
Delayed recall: accuracy (% correct). IM, intramuscular; IV, intravenous; PO, oral; SC, subcutaneous. *Studies that used microgram doses have been converted to milligrams based on a 75 kg body weight.

#### Digit span (Supplementary Figure S6)

Thirteen studies (24 strata; scopolamine *n* = 331, placebo *n* = 278) assessed digit span forward, while four studies (10 strata; scopolamine *n* = 157, placebo *n* = 119) assessed digit span backward. Scopolamine had no overall effect on the digit span forward (*G* = −0.158, 95% CI: −0.42 to 0.11, *p* = 0.239), although the injection group showed impairment (*G* = −0.29, 95% CI: −0.56 to −0.02, *p* = 0.034). Scopolamine impaired the digit span backward performance compared to placebo (*G* = −0.39, 95% CI: −0.68 to −0.09, *p* = 0.011). Comparing dose levels, digit span forward showed no significant effect at either high or low doses, while scopolamine impaired the performance at high doses but not at low doses for digit span backward (Supplementary Figure S7). No evidence of publication or reporting bias was observed (Supplementary Figure S8). Performance was measured across a large time duration (30 min–70 h) post-scopolamine administration with no clear impact of timing.

#### Buschke selective reminding task (Supplementary Figure S9)

Ten studies examined accuracy utilizing the Buschke selective reminding task (16 strata; scopolamine *n* = 225, placebo *n* = 173), while five studies (10 strata; scopolamine *n* = 137, placebo *n* = 85) investigated consistency. The scopolamine group performed worse than placebo on both accuracy (*G* = −1.13, 95% CI: −1.43 to −0.83, *p* < 0.001) and consistency tasks (*G* = −1.33, 95% CI: −1.8 to −0.86, *p* < 0.001). Scopolamine also significantly impaired accuracy and consistency at both high and low doses (Supplementary Figure S10). Evidence of publication or reporting bias was observed in the delayed recall task assessing performance (Supplementary Figure S11). This task was assessed 55 min–2.5 h post-scopolamine administration, with no distinguishable impact of time evident.

#### Recognition memory (Supplementary Figure S12)

For the recognition memory task, eight studies (19 strata; scopolamine *n* = 282, placebo *n* = 282) examined accuracy, while five studies (12 strata; scopolamine *n* = 208, placebo *n* = 208) investigated the reaction time. Scopolamine significantly impaired both accuracy (*G* = −0.43, 95% CI: −0.73 to −0.14, *p* = 0.004) and reaction time (*G* = 0.19, 95% CI: 0.001 to 0.37, *p* = 0.048) compared to the placebo group. High-dose scopolamine significantly impaired accuracy, while low-dose scopolamine had no effect (Supplementary Figure S13). This task was measured 30 min–4 h post-scopolamine administration, with no obvious impact of timing.

#### Sternberg memory scanning task (Supplementary Figure S14)

Four studies (five strata; scopolamine *n* = 89, placebo *n* = 89) utilized the Sternberg memory scanning task. Individuals in the scopolamine group performed worse on accuracy (*G* = −0.82, 95% CI: −1.27 to −0.38, *p* < 0.001) and had slower reaction times (*G* = 0.70, 95% CI: 0.20 to 1.20, *p* = 0.006) compared to placebo. This task was measured across 55 min–3 h post-scopolamine administration, although no observable impact of time was evident.

#### Age and sex

Age analysis was conducted for free/immediate recall, digit span forward, and the Buschke selective reminding task (Supplementary Figures S15–S17). Scopolamine impaired both young and old cohorts in free/immediate recall and the Buschke selective reminding task, but only affected the young cohort in digit span forward. There were insufficient studies to conduct a meaningful sex analysis.

### Attention

Five tasks provided measures of performance and reaction time. Scopolamine negatively impaired performance and significantly delayed reaction time during attention tasks, especially when post-administration scores were compared to baseline.

#### Choice reaction time (CRT) (Supplementary Figures S18–S21)

Twelve studies (27 strata; scopolamine *n* = 423, placebo *n* = 385) assessed reaction time, and four studies (eight strata; scopolamine *n* = 131, placebo *n* = 93) evaluated accuracy. The scopolamine group demonstrated a slower reaction time (*G* = 0.80, 95% CI: 0.48 to 1.13, *p* < 0.001), but not reduced accuracy (*G* = −0.5, 95% CI: −1.04 to 0.03, *p* = 0.063) compared to placebo. The effect size for a slower reaction time was larger for those who received scopolamine by injection (*G* = 1.25, 95% CI: 0.78 to 1.71, *p* < 0.001) compared to non-injectable scopolamine (*G* = 0.39, 95% CI: −0.06 to 0.84, *p* = 0.091). Comparing pre- to post-administration scores, seven studies (28 strata; scopolamine *n* = 259, placebo *n* = 221) investigated change in reaction time, and three studies (seven strata; scopolamine *n* = 114, placebo *n* = 76) examined change in accuracy. Scopolamine demonstrated slower reaction times (*G* = 2.08, 95% CI: 1.54 to 2.61, *p* < 0.001) and reduced accuracy (*G* = −0.86, 95% CI: −1.3 to −0.42, *p* < 0.001) compared to placebo, with the injection group demonstrating slower reaction times. Scopolamine impaired reaction time at both high and low doses compared to placebo and worsened reaction time from pre- to post-administration (Supplementary Figures S22 and S23). This task was measured 45 min–70 h post-administration, with no impact of timing. Publication or reporting bias was evident for the CRT task assessing reaction time (Supplementary Figure S24). The adjusted values with the imputed studies reduced the effect size from *G* = 0.83 to *G* = 0.64 (95% CI: 0.21−1.08, *p* = 0.004).

#### Simple reaction time (SRT) (Supplementary Figures S19 and S21)

Eight studies (13 strata; scopolamine *n* = 179, placebo *n* = 179) utilized the SRT task. The scopolamine group showed slower reaction times (*G* = 0.48, 95% CI: 0.15 to 0.81, *p* = 0.004) compared to placebo, with injectable administration demonstrating a larger effect size (*G* = 0.85, 95% CI: 0.22 to 1.56, *p* = 0.008) compared to the non-injection group (*G* = 0.34, 95% CI: −0.05 to 0.73, *p* = 0.083). Comparing pre- to post-administration scores, scopolamine was associated with slower reaction time (*G* = 0.88, 95% CI: 0.37 to 1.40, *p* = 0.001) compared to placebo. Comparing doses, scopolamine impaired reaction time at low doses but not at high doses (Supplementary Figure S25). Tasks ranged from 30 min to 4.5 h after scopolamine administration, with no apparent impact of time of administration.

#### Continuous performance task (CPT) (Supplementary Figure S19)

Four studies (10 strata; scopolamine *n* = 115, placebo *n* = 117) utilized the CPT (seven strata utilized an injectable mode of administration), with no significant effects of scopolamine compared to placebo. Measurements ranged from 1.5 to 70 h post-administration, with no impact of time of administration.

#### Rapid visual information processing (RVP) (Supplementary Figures S20 and S21)

Three studies (three strata (all injectable routes); scopolamine *n* = 48, placebo *n* = 48) utilized RVP. Examining change scores from baseline to post-scopolamine administration, scopolamine demonstrated slower reaction time (*G* = −1.16, 95% CI: −1.89 to −0.44, *p* = 0.002) and less accuracy (*G* = 1.74, 95% CI: 1.28 to 2.21, *p* < 0.001) compared to placebo. This task was measured 1–2 h post-scopolamine administration, with no impact of time of administration.

#### Vigilance task (Supplementary Figure S18)

Three studies (five strata; scopolamine *n* = 127, placebo *n* = 127, three strata used an injectable mode of administration) utilized the vigilance task. No differential effects of scopolamine compared to placebo were noted for this task. This task was measured across 1–15.5 h post-scopolamine administration, with no impact of the time of administration.

#### Age and sex

There were too few studies to conduct a meaningful age or sex analysis for attention tasks.

## Discussion

Scopolamine demonstrated a clear impairment for both memory and attention, particularly for tasks associated with working, episodic and recognition memory, and sustained attention utilizing this comprehensive systematic review and meta-analysis in healthy adults (Supplementary Tables S6 and S7). Similarly, scopolamine’s adverse impact on memory and attention was greater with an injectable method of administration (e.g., IV, IM, and SC) compared to non-injectable routes (e.g., PO, TD, and IN).

Despite some previous divergent findings [41], we believe the results of this systematic review support scopolamine administration in an injectable format as a useful model for cognitive dysfunction and dementia, with delayed recall (a working memory task), for example, noted as impaired in early-stage Alzheimer’s disease and clearly worsened by scopolamine administration [53, 54]. Furthermore, scopolamine-induced cognitive impairments are potentially relevant to understanding the cognitive deficits seen in schizophrenia, MDD and BD. The cholinergic system’s role in these psychiatric disorders is underscored by our findings that scopolamine can impact cognitive functions such as memory and attention, which are core components affected in these psychiatric disorders. These results not only support the hypothesis of cholinergic dysregulation in schizophrenia and MDD but also suggest that anticholinergic agents like scopolamine could potentially provide a valuable tool for investigating the neurochemical underpinnings of these conditions.

In comparison to placebo, scopolamine significantly impaired performance and consistency on the Buschke selective reminding test (Supplementary Figure S9), which evaluates the organization of long-term memory retrieval. Scopolamine also worsened performance and reaction times on the recognition memory (Supplementary Figure S12) and Sternberg tasks (Supplementary Figure S14), with the latter assessing working memory retrieval speed. While the digit span forward task (Supplementary Figure S6), a measure of working memory and attention, was not significantly affected, the scopolamine group did perform worse on this task. Scopolamine modestly impaired performance on the digit span backward task (Supplementary Figure S6), likely due to its lower difficulty compared to other working memory tasks (i.e., immediate and delayed recall) [55].

Similarly, the route of scopolamine administration affects its impact on cognitive performance in attention tasks. The injectable group exhibited slower reaction times on the CRT task compared to the non-injectable group (Supplementary Figure S19). The CRT task assesses sustained attention, and slower reaction times are indicative of poorer performance in attention tasks. Scopolamine also led to slower reaction times for both the SRT and RVP tasks (Supplementary Figure S21). Across all routes of administration, scopolamine negatively impacted performance on the CRT and RVP tasks compared to placebo (Supplementary Figure S20). There was no effect of scopolamine on the CPT and the vigilance task (Supplementary Figures S18 and S19); however, only three studies included these tasks, suggesting that the analysis may be underpowered to detect significant effects.

A likely rationale for the more significant cognitive deficits associated with injectable methods of scopolamine relate to its higher bioavailability with 100% absorption into the blood stream (half-life ~68.7 min) for IV scopolamine compared to 13% bioavailability (half-life ~63.7 min) for PO administration and even slower delivery for TD administration of (>4 h) [56]. PET imaging utilizing [11C] scopolamine further supports this by demonstrating that IV administration enables rapid CNS penetration and significant receptor occupancy, reflecting high bioavailability [1]. Therefore, methods with higher bioavailability, such as injectables, consequently have a greater impact on memory and attention than lower bioavailability.

The varied timing of task administration in this meta-analysis complicates conclusions about scopolamine’s impact on cognition. Cognitive deficits were observed as early as 1 h post-administration, but studies assessing memory 30–45 min post-administration found no significant effects [22, 41, 57, 58], and adverse effects were minimal after 6 h. For instance, free/immediate recall was unaffected after 6 h [28, 31], and digit span forward displayed no deficits compared to placebo at 22, 46, and 72 h [23]. Similarly, the CRT task displayed no effect on reaction time 30 min post-administration [59], with negligible effects for attention tasks evident after 11 h [23, 46]. These results should also be considered in the context of differing pharmacokinetic profiles associated with the route of administration. For example, injectable scopolamine achieves rapid systemic availability and peak effects, potentially explaining the early cognitive deficits observed, while PO or TD administration produces a slower onset of action with more sustained plasma concentrations. Consequently, although scopolamine, particularly when administered via injectable methods, impacts cognition, these effects are not long-lasting. This is of particular importance given the potential benefit IV scopolamine may impart for individuals experiencing a depressive episode [14, 15, 18].

Higher doses of scopolamine consistently impaired memory and attention, while lower doses also produced significant deficits in several tasks, particularly free/immediate recall, delayed recall, and CRT reaction time. However, some tasks, such as digit span forward, were unaffected, and in certain cases (e.g., digit span backward, recognition memory, and SRT reaction time), impairments were observed only at high or low doses, suggesting task-specific dose sensitivity. In addition, physiological factors such as body weight and gender may impact scopolamine’s pharmacokinetics. As scopolamine is highly lipid soluble, facilitating its redistribution into fatty tissues, gender (i.e., women generally have a higher fat content than men with a similar body mass index) and body weight may result in different distribution and clearance rates of scopolamine. Further research should consider body weight, sex differences, and other physiological variables. In addition, microgram doses have been converted to milligrams based on a 75 kg body weight for 11 studies, which potentially adds confounding variation to the analyses. While this approach helps standardize dosing, we acknowledge its limitations, as it may not fully account for individual differences in body composition and metabolism.

This study has other limitations. Older studies (pre-2000) had lower quality scores based on the Jadad rating scale, although all included trials were randomized and double-blinded [50]. Several studies fulfilling the inclusion criteria also had to be excluded due to insufficient extractable data. In addition, fewer studies evaluated certain memory and attention tasks, making comparisons between injectable and non-injectable administration methods unfeasible for some tasks. Moreover, an inadequate number of individual studies restricted the analysis of evidence for publication or reporting bias. However, where possible, consistency and precision across effects were examined.

In conclusion, this systematic review and meta-analysis, the largest to date investigating scopolamine’s effect on cognition in a healthy population, provides evidence of scopolamine’s negative effects on both memory and attention, with cognitive impairment more significant via injectable compared to non-injectable routes of administration. Despite scopolamine’s long-established use in medical practice, notable gaps persist in our understanding of its pharmacological impacts, especially its potential as a rapid antidepressant. Given the preliminary evidence supporting scopolamine’s use in treating depressive episodes, additional randomized controlled trials are suggested to determine optimal dosages and administration methods that maximize antidepressant benefits while minimizing adverse effects. Future clinical trials should evaluate the bioavailability of scopolamine across different routes of administration, to ensure its therapeutic benefits outweigh any potential adverse cognitive effects.

## Supporting information

Miravalles et al. supplementary material Miravalles et al. supplementary material

## Data Availability

Data are available upon request.

## Supplementary material

The supplementary material for this article can be found at http://doi.org/10.1192/j.eurpsy.2025.2446.
