# Supplementary material for: The effect of scopolamine on memory and attention: a systematic review and meta-analysis
Source: Eur Psychiatry. 2025 Apr 8;68(1):e50. doi: 10.1192/j.eurpsy.2025.2446 (PMC12041729; doi:10.1192/j.eurpsy.2025.2446)
Supplement: Miravalles et al. supplementary material [file S0924933825024460sup001.docx]

**SUPPLEMENTARY MATERIAL**

**Supplementary Table 1**. Pharmacokinetics Data of Scopolamine

| **Route** | **Dose**  **(mg)** | **C_max_**  **_(_ng/mL)** | **t_max_**  **(minutes)** | **AUC (ng*min/m)** | **F**  **(%)** | **CL**  **(L/h)** | **Vd**  **(L)** | **t½**  **(minutes)** |
| --- | --- | --- | --- | --- | --- | --- | --- | --- |
| **Oral** | 0.5 | 0.54± 0.1 | 23.5± 8.2 | 50.8± 1.76 | 13 ± 1 | ND | ND | 63.7± 1.3 |
| **IV** | 0.5 | 5.00±0.43 | 5 | 369.4±2.2 | 100 | 81.2± 1.55 | 141.3±1.6 l | 68.7± 1.0 |
| **SC** | 0.4 | 3.27 | 14.6 | 158.2 | ND | 0.14 -0.17 | ND | 213 |
| **IM** | 0.5 | 0.96± 0.17 | 18.5± 4.7 | 81.3 ± 11.2 | ND | ND | ND | 69.1± 8.0 |
| **IN** | 0.4 | 1.68± 0.23 | 2.2 ± 3 | 167.0 ± 20.0 | 83.0±10.0 | ND | ND | ND |
| **TD** | 1.5 | Approx. 0.1 | within 1140 | ND | ND | ND | ND | within 240 |

PO = oral, IV = intravenous, IM = intramuscular, SC = subcutaneous, IN = intranasal, TD = transdermal, ND = no data available, mg = milligram, C_max_ = Maximum plasma concentration, ng/mL = nanograms per milliliter, t_max_ = time taken by the drug to reach maximum concentration (C_max_), AUC = Area under curve, ng·min/mL = nanograms × minutes per milliliter, F = Absolute bioavailability, % = percentage, CL = clearance, L/h = liters per hour, Vd= volume of distribution, L = liter, t½ = half-life, Approx. = approximately

Data adapted from Renner et al., 2005

**Supplementary Table 2.** PRISMA Checklist (continued)

| **Section and Topic** | **Item #** | **Checklist item** | **Location where item is reported** |
| --- | --- | --- | --- |
| Synthesis methods | 13a | Describe the processes used to decide which studies were eligible for each synthesis (e.g. tabulating the study intervention characteristics and comparing against the planned groups for each synthesis (item #5)). | 6 |
|  | 13b | Describe any methods required to prepare the data for presentation or synthesis, such as handling of missing summary statistics, or data conversions. | 7 |
|  | 13c | Describe any methods used to tabulate or visually display results of individual studies and syntheses. | 7-8 |
|  | 13d | Describe any methods used to synthesize results and provide a rationale for the choice(s). If meta-analysis was performed, describe the model(s), method(s) to identify the presence and extent of statistical heterogeneity, and software package(s) used. | 7-8 |
|  | 13e | Describe any methods used to explore possible causes of heterogeneity among study results (e.g. subgroup analysis, meta-regression). | 8 |
|  | 13f | Describe any sensitivity analyses conducted to assess robustness of the synthesized results. | 7-8 |
| Reporting bias assessment | 14 | Describe any methods used to assess risk of bias due to missing results in a synthesis (arising from reporting biases). | 7 |
| Certainty assessment | 15 | Describe any methods used to assess certainty (or confidence) in the body of evidence for an outcome. | 8 |
| **RESULTS** | | |  |
| Study selection | 16a | Describe the results of the search and selection process, from the number of records identified in the search to the number of studies included in the review, ideally using a flow diagram. | 9, Fig. 1 |
|  | 16b | Cite studies that might appear to meet the inclusion criteria, but which were excluded, and explain why they were excluded. | 9, Fig. 1, Supp. Table 5 |
| Study characteristics | 17 | Cite each included study and present its characteristics. | Supp. Table 3 & 4 |
| Risk of bias in studies | 18 | Present assessments of risk of bias for each included study. | Supp. Table 3 & 4 |
| Results of individual studies | 19 | For all outcomes, present, for each study: (a) summary statistics for each group (where appropriate) and (b) an effect estimate and its precision (e.g. confidence/credible interval), ideally using structured tables or plots. | 10-16, Fig 2&3, Supp. Fig. 1-27 |
| Results of syntheses | 20a | For each synthesis, briefly summarise the characteristics and risk of bias among contributing studies. | 10-15, Supp. Fig. 3, 5, 8, 11, 24 |
|  | 20b | Present results of all statistical syntheses conducted. If meta-analysis was done, present for each the summary estimate and its precision (e.g. confidence/credible interval) and measures of statistical heterogeneity. If comparing groups, describe the direction of the effect. | 10-16, Fig 2-3, Supp. Fig. 1-27 |
|  | 20c | Present results of all investigations of possible causes of heterogeneity among study results. | 10-16, Fig 2-3, Supp. Fig. 1-27 |
|  | 20d | Present results of all sensitivity analyses conducted to assess the robustness of the synthesized results. | 10-16, Fig 2-3, Supp. Fig. 1-27 |

**Supplementary Table 2.** PRISMA Checklist (continued)

| **Section and Topic** | **Item #** | **Checklist item** | **Location where item is reported** |
| --- | --- | --- | --- |
| Reporting biases | 21 | Present assessments of risk of bias due to missing results (arising from reporting biases) for each synthesis assessed. | 10-15, Supp. Fig. 3, 5, 8, 11, 24 |
| Certainty of evidence | 22 | Present assessments of certainty (or confidence) in the body of evidence for each outcome assessed. | 10-16, Fig 2-3, Supp. Fig. 1-27 |
| **DISCUSSION** | | |  |
| Discussion | 23a | Provide a general interpretation of the results in the context of other evidence. | 16-19 |
|  | 23b | Discuss any limitations of the evidence included in the review. | 17-19 |
|  | 23c | Discuss any limitations of the review processes used. | 19 |
|  | 23d | Discuss implications of the results for practice, policy, and future research. | 19-20 |
| **OTHER INFORMATION** | | |  |
| Registration and protocol | 24a | Provide registration information for the review, including register name and registration number, or state that the review was not registered. | 1 |
|  | 24b | Indicate where the review protocol can be accessed, or state that a protocol was not prepared. | 5 |
|  | 24c | Describe and explain any amendments to information provided at registration or in the protocol. | No amendments |
| Support | 25 | Describe sources of financial or non-financial support for the review, and the role of the funders or sponsors in the review. | 20 |
| Competing interests | 26 | Declare any competing interests of review authors. | 20 |
| Availability of data, code and other materials | 27 | Report which of the following are publicly available and where they can be found: template data collection forms; data extracted from included studies; data used for all analyses; analytic code; any other materials used in the review. | Available upon request |

*From:*  Page MJ, McKenzie JE, Bossuyt PM, Boutron I, Hoffmann TC, Mulrow CD, et al. The PRISMA 2020 statement: an updated guideline for reporting systematic reviews. BMJ 2021;372:n71. doi: 10.1136/bmj.n71

**Supplementary Table 3.** Sociodemographic and Clinical Characteristics of Included Injection Studies

| Study | Age Range (mean) | Sex Percentages (M/F) | Scopolamine *n* | Placebo *n* | Route | Dose | Time after administration | JADAD (1-5) |
| --- | --- | --- | --- | --- | --- | --- | --- | --- |
| Dumas *et al*. 2008 (1) | 50-81 | 0/100 | 11 | 11 | Intravenous | 0.3mg* | 90 minutes | 5 |
| Potter *et al.* 2000 (2) | 19.8 | 30/70 | 12 | 12 | Intravenous | 0.7mg* | 30 minutes | 4 |
| Broocks *et al.* 1998 (3) | 21.8 | 40/60 | 10 | 10 | Intravenous | 0.4mg | 70 minutes | 4 |
| Little *et al.* 1998 (4) | 61.9 | 37.5/62.5 | 8 | 8 | Intravenous | 0.4mg | 75 minutes | 5 |
| Obonsawin *et al.* 1998 (5) | 40.8 | 25/75 | 12 | 12 | Intravenous | 0.5mg | 45 minutes | 4 |
| Martinez *et al.* 1997 (6) | 19-51 (28.4) | 65/35 | 17 | 17 | Intravenous | 0.5mg | 60-120 minutes | 4 |
| Vitiello *et al.* 1997 (7) | 20-33 (25) | 100/0 | 12 | 12 | Intravenous | 0.5mg | 30 minutes | 4 |
| Rabey *et al.* 1996 (8) | 67 | 45/55 | 9 | 9 | Intravenous | 0.5mg | 90 minutes | 4 |
| Tariot *et al.* 1996 (9) | 40-89 (63.2) | 35/65 | 19 | 19 | Intravenous | 0.1, 0.25, 0.5mg | 90 minutes | 4 |
| Molchan *et al.* 1992 (10) | Y: 27,  E: 66.5 | NA | Y: 46  E: 18 | Y: 46  E: 18 | Intravenous | 0.5mg | 90 min | 4 |
| Kopelman and Corn 1988 (11) | 38.2 | 40/60 | 10 | 10 | Intravenous | 0.2, 0.4mg | 30 min | 2 |
| Newhouse *et al.* 1988 (12) | 62-78 (69.7) | 66.6/33.3 | 9 | 9 | Intravenous | 0.1, 0.25, 0.5mg | 90 min | 4 |

**Supplementary Table 3.** Continued

| Study | Age Range (mean) | Sex Percentages (M/F) | Scopolamine *n* | Placebo *n* | Route | Dose | Time After Administration | JADAD (1-5) |
| --- | --- | --- | --- | --- | --- | --- | --- | --- |
| Sunderland *et al.* 1987 (13) | 61.3 | 30/70 | 10 | 10 | Intravenous | 0.1, 0.25, 0.5mg | 90 min | 4 |
| Petersen 1979 (14) | 18-28 (20.93) | 100/0 | 7 | 7 | Intravenous | 0.6mg* | 45 min | 2 |
| Bartholomeusz et al. 2008 (15) | 18-38 (22.4) | 0/100 | 14 | 14 | Intramuscular | 0.4mg | 90 minutes | 5 |
| Ellis *et al.* 2006 (16) | 19-27 (22.4) | 83.3/16.6 | 12 | 12 | Intramuscular | 0.4mg | 120 minutes | 4 |
| Meador *et al.* 1995 *(17)* | 24-40 (26) | 55/45 | 11 | 11 | Intramuscular | 0.5mg* | 120 minutes | 4 |
| Lines *et al*. 1993 (18) | 21-45 (28) | 100/0 | 24 | 24 | intramuscular | 0.4mg | 30 min | 4 |
| Canal *et al.* 1991 (19) | 19-38 (26.5) | 56/44 | 8 | 8 | Intramuscular | 0.5mg | 30, 60, 120, 180, 360 min | 4 |
| Danion *et al.* 1990 (20) | 20-27 (23) | NA | 12 | 12 | Intramuscular | 0.75mg* | 60 min | 4 |
| Meador *et al.* 1988 (21) | 21-43 (30) | 62.5/37.5 | 12 | 12 | Intramuscular | 0.9mg* | 55 min | 4 |
| Ghoneim and Mewaldt 1977 (22) | 19-32 (23) | 50/50 | 10 | 10 | Intramuscular | 1mg* | 30-115 min | 4 |
| Ghoneim and Mewaldt 1975 (23) | 19-27 (21) | 50/50 | 9 | 9 | Intramuscular | 1mg* | 35-110 min | 4 |
| Mintzer and Griffiths 2007 (24) | 19-43 (27) | 45/55 | 20 | 20 | Subcutaneous | 0.25, 0.5mg | 55 minutes | 4 |

**Supplementary Table 3.** Continued

| Study | Age Range (mean) | Sex Percentages (M/F) | Scopolamine *n* | Placebo *n* | Route | Dose | Time after administration | JADAD (1-5) |
| --- | --- | --- | --- | --- | --- | --- | --- | --- |
| Koller *et al.* 2003 (25) | 23.8 | 33.3/66.6 | 12 | 12 | Subcutaneous | 0.3, 0.6mg | 90-180 minutes | 4 |
| Bédard *et al.* 1999 (26) | 68.1 | NA | 10 | 10 | Subcutaneous | 0.25mg | 30 minutes | 2 |
| Ebert *et al.* 1998 (27) | 22-25 (24) | 100/0 | 10 | 10 | Subcutaneous | 0.4, 0.6, 0.8mg | 1, 2, 3, 5, and 8 hours | 5 |
| Robbins *et al.* 1997 (28) | 29 | 100/0 | 24 | 24 | Subcutaneous | 0.2, 0.4, 0.6mg | 90 minutes | 4 |
| Duka *et al.* 1996 (29) | 21-37 | NA | 18 | 18 | Subcutaneous | 0.5mg | 120-300 minutes | 4 |
| Riedel *et al.* 1995 (30) | 25-35 | 50/50 | 18 | 18 | Subcutaneous | 0.5mg* | 2, 4 and 6 hr | 5 |
| Dunne *et al.* 1993 (31) | 22-59 (36.4) | 100/0 | 10 | 10 | Subcutaneous | 0.4mg | 45 min | 2 |
| Flicker *et al.* 1992 *(32)* | Y: 18-30 (23.1),  E: 60-85 (70) | Y: 60/40  E: 30/70 | 10 | 10 | Subcutaneous | 0.5mg* | 30 min | 0 |
| Knopman 1991 (33) | 18-33 | 50/50 | 13 | 12 | Subcutaneous | 0.5mg* | 90 min | 4 |
| Patat *et al.* 1991 (34) | 19-30 (25) | 100/0 | 12 | 12 | Subcutaneous | 0.6mg | 60-150 min | 4 |
| Wesnes *et al.* 1988 (35) | 21.1 | 100/0 | 18 | 18 | Subcutaneous | 0.6mg | 60 min | 4 |

The Jadad scale was used to assess the reliability and validity of studies. This tool assesses randomization, blinding, and study withdrawals on a 5-point scale.

*Studies that used microgram doses have been converted to milligrams based off 75kg body weight.

Y = young cohort

E = elderly cohort

NA = data not attainable

**Supplementary Table 4.** Sociodemographic and Clinical Characteristics of Included Non-injection Studies

| Study | Age Range (mean) | Sex Percentages (M/F) | Scopolamine *n* | Placebo *n* | Route | Dose | Time after administration | JADAD (1-5) |
| --- | --- | --- | --- | --- | --- | --- | --- | --- |
| Smyth et al. 2019 (36) | 24.2 | 50/50 | 18 | 18 | Oral | 0.6mg | 120 minutes | 2 |
| Golding et al. 2018 (37) | 17-43 (26.47) | 100/0 | 15 | 15 | Oral | 0.6mg | 1.5, 3.5 and 4.5 hours | 4 |
| Van Ruitenbeek *et al.* 2008 (38) | 18-45 (21.6) | 0/100 | 20 | 20 | Oral | 1mg | 2 & 4 hours | 3 |
| Rammsayer *et al.* 2000 (39) | 20-35 (25.3) | 100/0 | 20 | 20 | Oral | 1mg | 60 minutes | 4 |
| Broks *et al.* 1988 (40) | 18-48 | 50/50 | 20 | 20 | Oral | 0.3, 0.6, 1.2mg | 45-60 min | 2 |
| Schmedtje *et al.* 1988 (41) | NA | NA | 8 | 8 | Oral | 0.5mg | 150-180 min | 4 |
| Dunne and Hartley 1985 (42) | NA | 0/100 | 47 | 47 | Oral | 0.6mg | 90 min | 4 |
| Brazell *et al.* 1989 (43) | 20-29 | 58/42 | 12 | 12 | Transdermal | 60pg/ml | 22, 46 and 70 hr | 2 |
| Gordon *et al.* 1986 (44) | 18-20 | 100/0 | 23 | 23 | Transdermal | 0.2mg | 11-15.5 h | 4 |
| Weerts et al. 2015 (45) | 24.4 | 100/0 | 19 | 19 | Intranasal | 0.4mg | 180 minutes | 4 |

The Jadad scale was used to assess the reliability and validity of studies. This tool assesses randomization, blinding, and study withdrawals on a 5-point scale.

NA = data not attainable

**Supplementary Table 5.** Unique Cognitive Tasks

| **Cognitive Task** | **Studies** |
| --- | --- |
| *Memory* | |
| 1. Auditory verbal learning test | Bukala *et a*l., 2019; Koller *et al.,* 2023 |
| 1. Baddeley reasoning test | **Duka *et al*., 1996**; Curran *et al.,* 1991 |
| 1. Benton visual retention test | Dumas *et al*., 2006; **Dunne *et al*., 1993** |
| 1. Block span | **Vitiello *et al*., 1997**; **Kopelman *et al*., 1998** |
| 1. Complex figures | **Smyth *et al.,* 2019**; Meador *et al.,* 1993 |
| 1. Consistent long-term retrieval | **Patat *et al.,* 1991; Meador *et al.,* 1988** |
| 1. Cued recall (episodic memory) | Mintzer *et al.,* 2010; **Bedard *et al.,*1999** |
| 1. Delayed matching to sample | Reches *et al*., 2014; **Robbins *et al.,* 1997** |
| 1. Delayed Groton Maze Learning Test | Cho *et al.,* 2011; Fredrickson *et al.,* 2008 |
| 1. Groton Maze Learning Test* | Thomas *et al*., 2008; Snyder *et al.,* 2005 |
| 1. Matching to sample | Koller *et al*., 2003; **Robbins *et al*., 1997** |
| 1. NYU paragraph recall | **Dumas *et al*., 2008**; Duma *et al*., 2006 |
| 1. Object naming task | **Broocks *et al.,* 1998**; Little *et al.,* 1995 |
| 1. Paired associates learning | Harel *et al.,* 2013; **Robbins *et al.,* 1997** |
| 1. Paragraph story | Meador *et al*., 1993; **Flicker *et al.,* 1992** |
| 1. Processing speed | Little *et al.,* 1995; **Broocks *et al.,* 1998** |
| 1. Rivermead behavioral memory test | Kamboj *et al,* 2006; Schifano *et al.,* 1994 |
| 1. Spatial span | **Brazell *et al*., 1989; Broks *et al*., 1988** |
| 1. Visual memory test | **Duka *et al.,* 1996**; Brass *et al.,* 1995 |
| 1. Word fluency | Curran *et al.,* 1991; **Knopman *et al.,* 1991** |
| 1. Working memory** | **Mintzer *et al.,* 2007**; Furey *et al*., 2013 |
| *Attention* | |
| 1. Auditory sustained attention | **Lines *et al*., 1993; Brazell *et al.,* 1989** |
| 1. D2 concentration test | Voss *et al*., 2010; **Wesnes *et al.,* 1988** |
| 1. Digit cancellation test | Curran *et al*., 1991; **Canal *et al*., 1991** |
| 1. Trail-making task | **Tariot *et al.,* 1996**; Wesnes *et al.,* 1990 |
| 1. Visual sustained attention | **Lines *et al.,* 1993; Brazell *et al.,* 1989** |

*examined executive functioning rather than memory or attention; could not include.

**name of task was unspecified and did not align with any other memory task

This table demonstrates the list of unique cognitive tasks that could not be included since there were only 2 studies utilizing the task.

Studies in bold were already included in the meta-analysis.

**Supplementary Figure 1.** Free/Immediate & Delayed Recall – Change in Accuracy (% correct)

PO = oral, IM = intramuscular, IV = intravenous, SC = subcutaneous

*Studies that used microgram doses have been converted to milligrams based off a 75kg body weight.

**Supplementary Figure 2.** Free/immediate Recall: Accuracy (% correct) – Dose Comparisons

■ Old cohort.

□ Young cohort.

PO = oral, IM = intramuscular, IV = intravenous, SC = subcutaneous

*Studies that used microgram doses have been converted to milligrams based off a 75kg body weight.

**Supplementary Figure 3.** Publication or reporting bias in studies examining the Free/Immediate Recall task following scopolamine relative to placebo.

**
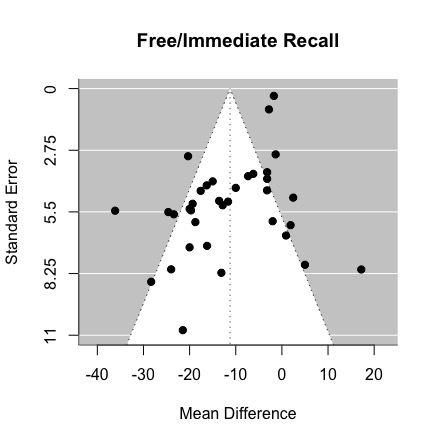
**

Standard error of the mean difference between scopolamine and placebo groups in performance in the free/immediate recall task.

**Supplementary Figure 4.** Delayed Recall: Accuracy (% correct) – Dose Comparisons

PO = oral, IM = intramuscular, IV = intravenous, SC = subcutaneous

*Studies that used microgram doses have been converted to milligrams based off a 75kg body weight.

**Supplementary Figure 5.** Publication or reporting bias in studies examining the Delayed Recall task following scopolamine relative to placebo.


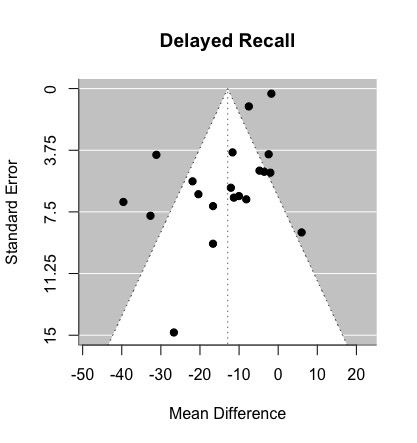


Standard error of the mean difference between scopolamine and placebo groups in performance in the delayed recall task.

**Supplementary Figure 6.** Digit Span

■ Old cohort.

□ Young cohort.

PO = oral, IM = intramuscular, IV = intravenous, SC = subcutaneous, TD = transdermal

*Studies that used microgram doses have been converted to milligrams based off a 75kg body weight.

**Supplementary Figure 7.** Digit Span – Dose Comparison

■ Old cohort.

□ Young cohort.

PO = oral, IM = intramuscular, IV = intravenous, SC = subcutaneous

*Studies that used microgram doses have been converted to milligrams based off a 75kg body weight.

**Supplementary Figure 8.** Publication or reporting bias in studies examining the Digit Span task following scopolamine relative to placebo.


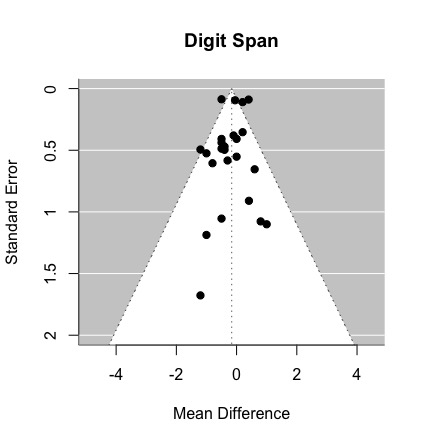


Standard error of the mean difference between scopolamine and placebo groups in performance in the digit span forward task.

**Supplementary Figure 9.** Buschke Selective Reminding Task

■ Old cohort.

□ Young cohort.

IM = intramuscular, IV = intravenous, PO = oral, SC = subcutaneous

*Studies that used microgram doses have been converted to milligrams based off a 75kg body weight.

**Supplementary Figure 10**. Buschke Selective Reminding Task – Dose Comparisons

■ Old cohort.

□ Young cohort.

IM = intramuscular, IV = intravenous, PO = oral, SC = subcutaneous

*Studies that used microgram doses have been converted to milligrams based off a 75kg body weight.

**Supplementary Figure 11.** Publication or reporting bias in studies examining the Buschke Selective Reminding Task following scopolamine relative to placebo.

**
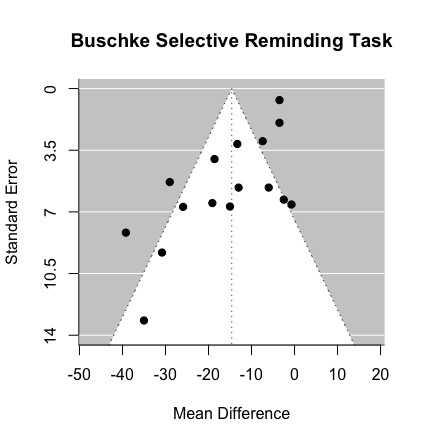
**

Standard error of the mean difference between scopolamine and placebo groups in performance in the Buschke Selective Reminding task.

**Supplementary Figure 12.** Recognition Memory Task

■Uses a pattern recognition memory task.

□Uses a spatial recognition memory task.

●Uses a word recognition memory task.

○Uses a picture recognition memory task.

IM = intramuscular, IV = intravenous, PO = oral, SC = subcutaneous

*Studies that used microgram doses have been converted to milligrams based off a 75kg body weight.

**Supplementary Figure 13.** Recognition Memory: Accuracy (% correct) – Dose Comparisons

■Uses a pattern recognition memory task.

□Uses a spatial recognition memory task.

●Uses a word recognition memory task.

○Uses a picture recognition memory task.

PO = oral, IM = intramuscular, IV = intravenous, SC = subcutaneous

*Studies that used microgram doses have been converted to milligrams based off a 75kg body weight.

**Supplementary Figure 14.** Sternberg Memory Scanning Task

IM = intramuscular, IN = intranasal, SC = subcutaneous

**Supplementary Figure 15.** Free/immediate Recall: Accuracy (% correct) – Age Analysis

■ Old cohort.

□ Young cohort.

PO = oral, IM = intramuscular, IV = intravenous, SC = subcutaneous

*Studies that used microgram doses have been converted to milligrams based off a 75kg body weight.

**Supplementary Figure 16.** Digit Span Forward – Age Analysis

■ Old cohort.

□ Young cohort.

IM = intramuscular, IV = intravenous, SC = subcutaneous, TD = transdermal

*Studies that used microgram doses have been converted to milligrams based off a 75kg body weight.

**Supplementary Figure 17.** Buschke Selective Reminding Task Accuracy (% correct) – Age Analysis

■ Old cohort.

□ Young cohort.

PO = oral, IM = intramuscular, IV = intravenous, SC = subcutaneous

**Supplementary Figure 18.** Cognitive Tasks for Attention: Accuracy (% correct)

CRT – Choice Reaction Time

PO = oral, IM = intramuscular, IV = intravenous, TD = transdermal

■ Old cohort.

□ Young cohort.

**Supplementary Figure 19.** Cognitive Tasks for Attention: Reaction Time (ms)

CRT – Choice Reaction Time; SRT – Simple Reaction Time; CPT – Continuous Performance Task

■ Old cohort.

□ Young cohort.

PO = oral, IM = intramuscular, IV = intravenous, SC = subcutaneous, TD = transdermal; pg/ml = pictograms per milliliter

*Studies that used microgram doses have been converted to milligrams based off a 75kg body weight.

**Supplementary Figure 20.** Cognitive Tasks for Attention: Change in Accuracy (%)

CRT – Choice Reaction Time; RVP – Rapid Visual Information Processing Task

PO = oral, IM = intramuscular, IV = intravenous, SC = subcutaneous

**Supplementary Figure 21.** Cognitive Tasks for Attention: Change in Reaction Time (ms)

CRT – Choice Reaction Time; SRT – Simple Reaction Time; RVP – Rapid Visual Information Processing Task

PO = oral, IM = intramuscular, IV = intravenous, SC = subcutaneous, TD = transdermal; pg/ml = pictograms per milliliter

**Supplementary Figure 22.** Choice Reaction Time: Reaction Time (ms) – Dose Comparisons

**■** Old cohort.

□ Young cohort.

PO = oral, IM = intramuscular, IV = intravenous, SC = subcutaneous

**Supplementary Figure 23.** Choice Reaction Time: Change in Reaction Time (ms) – Dose Comparisons

PO = oral, IM = intramuscular, IV = intravenous, SC = subcutaneous

**Supplementary Figure 24.** Publication or reporting bias in studies examining Choice Reaction Time (CRT) following scopolamine relative to placebo.


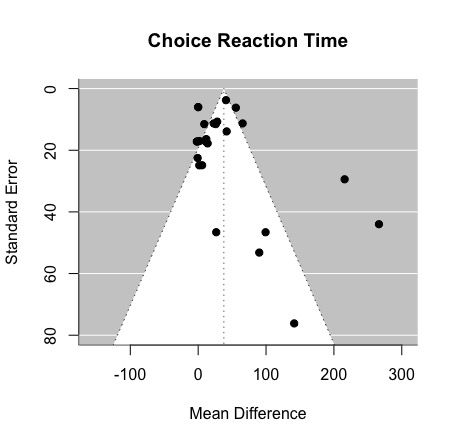


Standard error of the mean difference between scopolamine and placebo groups in reaction time in the CRT task.

**Supplementary Figure 25.** Simple Reaction Time: Reaction Time (ms) – Dose Comparisons

PO = oral, IM = intramuscular, IV = intravenous

**Supplementary Table 6.** Summary of Scopolamine’s Effect on Memory (*P*-Values Reported)

| **Group** | **Measurement** | **Free/Immediate Recall** | **Delayed Recall** | **Digit Span Forward** | **Digit Span Backward** | **BSRT** | **BSRT Consistency** | **Recognition Memory** | **SMST** |
| --- | --- | --- | --- | --- | --- | --- | --- | --- | --- |
| Overall | Accuracy/performance | <0.001* | <0.001* | 0.239 | 0.011* | <0.001* | <0.001* | 0.004* | <0.001* |
|  | Change in accuracy | <0.001* | 0.47 | - | - | - | - | - | - |
|  | Reaction time | - | - | - | - | - | - | 0.048* | 0.006* |
| Injection | Accuracy/performance | <0.001* | <0.001* | - | - | - | - | - | - |
| Non-Injection | Accuracy/performance | 0.566 | 0.018* | - | - | - | - | - | - |
| High Dose | Accuracy/performance | <0.001* | <0.001* | 0.239 | 0.001* | <0.001* | <0.001* | 0.001* | - |
| Low Dose | Accuracy/performance | 0.049* | <0.001* | 0.669 | 0.317 | <0.001* | 0.003* | 0.551 | - |
| Old Cohort | Accuracy/performance | 0.029* | - | 0.315 | - | <0.001* | - | - | - |
| Young Cohort | Accuracy/performance | <0.001* | - | 0.013* | - | <0.001* | - | - | - |

**p* < 0.05

BSRT = Buschke Selective Reminding Task; SMST = Sternberg Memory Scanning Task

**Supplementary Table 7.** Summary of Scopolamine’s Effect on Attention (*P*-Values Reported)

| **Group** | **Measurement** | **Choice Reaction Time** | **Simple Reaction Time** | **Continuous Performance Task** | **RVP** | **Vigilance Task** |
| --- | --- | --- | --- | --- | --- | --- |
| Overall | Accuracy/performance | 0.063 | - | - | - | 0.423 |
|  | Change in accuracy | <0.001* | - | - | 0.02* | - |
|  | Reaction time | <0.001* | 0.004* | 0.185 | - | - |
|  | Change in reaction time | <0.001* | 0.001* | - | <0.001* | - |
| Injection | Reaction time | <0.001* | 0.008* | - | - | - |
|  | Change in reaction time | <0.001* | - | - | - | - |
| Non-Injection | Reaction time | 0.091 | 0.083 | - | - | - |
|  | Change in reaction time | 0.552 | - | - | - | - |
| High dose | Reaction time | 0.001* | 0.098 | - | - | - |
|  | Change in reaction time | <0.001* | - | - | - | - |
| Low dose | Reaction time | 0.001* | 0.009* | - | - | - |
|  | Change in reaction time | <0.001* | - | - | - | - |

**p* < 0.05

RVP = Rapid Visual Information Processing

References

1. Dumas J, Hancur-Bucci C, Naylor M, Sites C, Newhouse P (2008): Estradiol interacts with the cholinergic system to affect verbal memory in postmenopausal women: evidence for the critical period hypothesis. *Horm Behav*. 53:159-169.

2. Potter DD, Pickles CD, Roberts RC, Rugg MD (2000): Scopolamine impairs memory performance and reduces frontal but not parietal visual P3 amplitude. *Biol Psychol*. 52:37-52.

3. Broocks A, Little JT, Martin A, Minichiello MD, Dubbert B, Mack C, et al. (1998): The influence of ondansetron and m-chlorophenylpiperazine on scopolamine-induced cognitive, behavioral, and physiological responses in young healthy controls. *Biol Psychiatry*. 43:408-416.

4. Little JT, Johnson DN, Minichiello M, Weingartner H, Sunderland T (1998): Combined nicotinic and muscarinic blockade in elderly normal volunteers: cognitive, behavioral, and physiologic responses. *Neuropsychopharmacology*. 19:60-69.

5. Obonsawin MC, Robertson A, Crawford JR, Perera C, Walker S, Blackmore L, et al. (1998): Non-mnestic cognitive function in the scopolamine model of Alzheimer's disease. *Human Psychopharmacology*. 13:439-449.

6. Martinez R, Molchan SE, Lawlor BA, Thompson K, Martinson H, Latham G, et al. (1997): Minimal effects of dextroamphetamine on scopolamine-induced cognitive impairments in humans. *Biol Psychiatry*. 41:50-57.

7. Vitiello B, Martin A, Hill J, Mack C, Molchan S, Martinez R, et al. (1997): Cognitive and behavioral effects of cholinergic, dopaminergic, and serotonergic blockade in humans. *Neuropsychopharmacology*. 16:15-24.

8. Rabey JM, Neufeld MY, Treves TA, Sifris P, Korczyn AD (1996): Cognitive effects of scopolamine in dementia. *J Neural Transm (Vienna)*. 103:873-881.

9. Tariot PN, Patel SV, Cox C, Henderson RE (1996): Age-related decline in central cholinergic function demonstrated with scopolamine. *Psychopharmacology (Berl)*. 125:50-56.

10. Molchan SE, Martinez RA, Hill JL, Weingartner HJ, Thompson K, Vitiello B, et al. (1992): Increased cognitive sensitivity to scopolamine with age and a perspective on the scopolamine model. *Brain Res Brain Res Rev*. 17:215-226.

11. Kopelman MD, Corn TH (1988): Cholinergic 'blockade' as a model for cholinergic depletion. A comparison of the memory deficits with those of Alzheimer-type dementia and the alcoholic Korsakoff syndrome. *Brain*. 111 ( Pt 5):1079-1110.

12. Newhouse PA, Sunderland T, Tariot PN, Weingartner H, Thompson K, Mellow AM, et al. (1988): The effects of acute scopolamine in geriatric depression. *Arch Gen Psychiatry*. 45:906-912.

13. Sunderland T, Tariot PN, Cohen RM, Weingartner H, Mueller EA, 3rd, Murphy DL (1987): Anticholinergic sensitivity in patients with dementia of the Alzheimer type and age-matched controls. A dose-response study. *Arch Gen Psychiatry*. 44:418-426.

14. Harel BT, Pietrzak RH, Snyder PJ, Maruff P (2013): Effect of cholinergic neurotransmission modulation on visual spatial paired associate learning in healthy human adults. *Psychopharmacology (Berl)*. 228:673-683.

15. Bartholomeusz CF, Wesnes KA, Kulkarni J, Vitetta L, Croft RJ, Nathan PJ (2008): Estradiol treatment and its interaction with the cholinergic system: effects on cognitive function in healthy young women. *Horm Behav*. 54:684-693.

16. Ellis JR, Ellis KA, Bartholomeusz CF, Harrison BJ, Wesnes KA, Erskine FF, et al. (2006): Muscarinic and nicotinic receptors synergistically modulate working memory and attention in humans. *Int J Neuropsychopharmacol*. 9:175-189.

17. Meador KJ, Loring DW, Hendrix N, Nichols ME, Oberzan R, Moore EE (1995): Synergistic anticholinergic and antiserotonergic effects in humans. *J Clin Exp Neuropsychol*. 17:611-621.

18. Lines CR, Ambrose JH, Heald A, Traub M (1993): A double-blind, placebo-controlled study of the effects of eptastigmine on scopolamine-induced cognitive deficits in healthy male subjects. *Human Psychopharmacology*. 8:271-278.

19. Canal N, Franceschi M, Alberoni M, Castiglioni C, De Moliner P, Longoni A (1991): Effect of L-alpha-glyceryl-phosphorylcholine on amnesia caused by scopolamine. *Int J Clin Pharmacol Ther Toxicol*. 29:103-107.

20. Danion JM, Zimmermann MA, Willard-Schroeder D, Grange D, Welsch M, Imbs JL, et al. (1990): Effects of scopolamine, trimipramine and diazepam on explicit memory and repetition priming in healthy volunteers. *Psychopharmacology (Berl)*. 102:422-424.

21. Meador KJ, Loring DW, Lee GP, Taylor HS, Hughes DR, Feldman DS (1988): In vivo probe of central cholinergic systems. *J Gerontol*. 43:M158-162.

22. Ghoneim MM, Mewaldt SP (1977): Studies on human memory: the interactions of diazepam, scopolamine, and physostigmine. *Psychopharmacology (Berl)*. 52:1-6.

23. Ghoneim MM, Mewaldt SP (1975): Effects of diazepam and scopolamine on storage, retrieval and organizational processes in memory. *Psychopharmacologia*. 44:257-262.

24. Mintzer MZ, Griffiths RR (2007): Differential effects of scopolamine and lorazepam on working memory maintenance versus manipulation processes. *Cogn Affect Behav Neurosci*. 7:120-129.

25. Koller G, Satzger W, Adam M, Wagner M, Kathmann N, Soyka M, et al. (2003): Effects of scopolamine on matching to sample paradigm and related tests in human subjects. *Neuropsychobiology*. 48:87-94.

26. Bedard MA, Pillon B, Dubois B, Duchesne N, Masson H, Agid Y (1999): Acute and long-term administration of anticholinergics in Parkinson's disease: specific effects on the subcortico-frontal syndrome. *Brain Cogn*. 40:289-313.

27. Ebert U, Siepmann M, Oertel R, Wesnes KA, Kirch W (1998): Pharmacokinetics and pharmacodynamics of scopolamine after subcutaneous administration. *J Clin Pharmacol*. 38:720-726.

28. Robbins TW, Semple J, Kumar R, Truman MI, Shorter J, Ferraro A, et al. (1997): Effects of scopolamine on delayed-matching-to-sample and paired associates tests of visual memory and learning in human subjects: comparison with diazepam and implications for dementia. *Psychopharmacology (Berl)*. 134:95-106.

29. Duka T, Ott H, Rohloff A, Voet B (1996): The effects of a benzodiazepine receptor antagonist beta-carboline ZK-93426 on scopolamine-induced impairment on attention, memory and psychomotor skills. *Psychopharmacology (Berl)*. 123:361-373.

30. Riedel W, Hogervorst E, Leboux R, Verhey F, van Praag H, Jolles J (1995): Caffeine attenuates scopolamine-induced memory impairment in humans. *Psychopharmacology (Berl)*. 122:158-168.

31. Dunne MP, Statham D, Raphael B, Kemp R, Kelly B (1993): Further evidence that scopolamine can improve verbal fluency. *Journal of Psychopharmacology*. 7:159-163.

32. Flicker C, Ferris SH, Serby M (1992): Hypersensitivity to scopolamine in the elderly. *Psychopharmacology (Berl)*. 107:437-441.

33. Knopman D (1991): Unaware learning versus preserved learning in pharmacologic amnesia: similarities and differences. *J Exp Psychol Learn Mem Cogn*. 17:1017-1029.

34. Patat A, Klein MJ, Surjus A, Hucher M, Granier J (1991): RU 41,656 does not reverse the scopolamine-induced cognitive deficit in healthy volunteers. *Eur J Clin Pharmacol*. 41:225-231.

35. Wesnes K, Simpson, P. and Kidd, A. (1988): An investigation of the range of cognitive impairments induced by scopolamine 0· 6 mg sc. *Human Psychopharmacology: Clinical and Experimental*. 3:27-41.

36. Smyth SF, Beversdorf DQ (2019): Muscarinic and Nicotinic Modulation of Memory but not Verbal Problem-solving. *Cognitive and Behavioral Neurology*. 32:278-283.

37. Golding JF, Wesnes KA, Leaker BR (2018): The effects of the selective muscarinic M3 receptor antagonist darifenacin, and of hyoscine (scopolamine), on motion sickness, skin conductance & cognitive function. *Br J Clin Pharmacol*. 84:1535-1543.

38. Van Ruitenbeek P, Vermeeren A, Riedel WJ (2008): Histamine H1-receptor blockade in humans affects psychomotor performance but not memory. *Journal of Psychopharmacology*. 22:663-672.

39. Rammsayer TH, Rodewald S, Groh D (2000): Dopamine-antagonistic, anticholinergic, and GABAergic effects on declarative and procedural memory functions. *Brain Res Cogn Brain Res*. 9:61-71.

40. Broks P, Preston GC, Traub M, Poppleton P, Ward C, Stahl SM (1988): Modelling dementia: effects of scopolamine on memory and attention. *Neuropsychologia*. 26:685-700.

41. Schmedtje JF, Oman CM, Letz R, Baker EL (1988): Effects of scopolamine and dextroamphetamine on human performance. *Aviation Space and Environmental Medicine*. 59:407-410.

42. Dunne MP, Hartley LR (1985): The effects of scopolamine upon verbal memory: evidence for an attentional hypothesis. *Acta Psychol (Amst)*. 58:205-217.

43. Brazell C, Preston GC, Ward C, Lines CR, Traub M (1989): The scopolamine model of dementia: chronic transdermal administration. *J Psychopharmacol*. 3:76-82.

44. Gordon C, Binah O, Attias J, Rolnick A (1986): Transdermal scopolamine: human performance and side effects. *Aviat Space Environ Med*. 57:236-240.

45. Weerts AP, Pattyn N, Putcha L, Hoag SW, Van Ombergen A, Hallgren E, et al. (2015): Restricted sedation and absence of cognitive impairments after administration of intranasal scopolamine. *Journal of Psychopharmacology*. 29:1231-1235.
